# Total Phenolic Content and Antioxidant Activity of Some Malvaceae Family Species

**DOI:** 10.3390/antiox1010033

**Published:** 2012-10-26

**Authors:** Adriana Maria Fernandes de Oliveira, Lilian Sousa Pinheiro, Charlane Kelly Souto Pereira, Wemerson Neves Matias, Roosevelt Albuquerque Gomes, Otemberg Souza Chaves, Maria de Fátima Vanderlei de Souza, Reinaldo Nóbrega de Almeida, Temilce Simões de Assis

**Affiliations:** 1 Universidade Federal de Campina Grande, Unidade Acadêmica de Ciências da Vida, CEP 58900-000, Cajazeiras, PB, Brazil; 2 Programa de Pós-Graduação em Produtos Naturais e Sintéticos Bioativos, Centro de Ciências da Saúde, Universidade Federal da Paraíba, CEP 58051-970, João Pessoa-PB, Brazil; E-Mails: lilihaaa@hotmail.com (L.S.P.); charlane_kelly@yahoo.com.br (C.K.S.P.); wnmathias21@yahoo.com.br (W.N.M.); roosevelt_ag@bol.com.br (R.A.G.); otemberg_sc@yahoo.com.br (O.S.C.); mfvanderlei@ltf.ufpb.br (M.F.V.S.); reinaldoan@uol.com.br (R.N.A.); 3 Departamento de Fisiologia e Patologia, Centro de Ciências da Saúde, Universidade Federal da Paraíba, CEP 58059-900, João Pessoa-PB, Brazil; E-Mail: temilce@yahoo.com.br

**Keywords:** Malvaceae family, antioxidant activity, total phenolic content, DPPH assay, TEAC assay

## Abstract

The antioxidant activity of four species of the Malvaceae family (*Sidastrum micranthum* (A. St.-Hil.) Fryxell, *Wissadula periplocifoli*a (L.) C. Presl, *Sida rhombifolia* (L.) E. H. L and *Herissantia crispa* L. (Brizicky)) were studied using the total phenolic content, DPPH radical scavenging activity and Trolox equivalent antioxidant capacity (TEAC) assays. The antioxidant activity of the crude extract, phases and two isolated flavonoids, kaempferol 3,7-di-O-α-L-rhamnopyranoside (lespedin) and kaempferol 3-O-β-D-(6*''*-E-p-coumaroil) glucopyranoside (tiliroside) was determined. The results showed that there is a strong correlation between total polyphenol contents and antioxidant activity of the crude extract of *Sidastrum micranthum* and *Wissadula periplocifolia*; however, this was not observed between *Sida rhombifolia* and *Herissantia crispa*. The ethyl acetate (EaF) phase showed the best antioxidant effect in the total phenolics, DPPH and TEAC assays, followed by the chloroform (CfF) phase, in most species tested. Lespedin, isolated from the EaF phase of *W. periplocifolia* and *H. crispa* may not be responsible for the antioxidant activity due to its low antioxidant activity (IC_50_: DPPH: 1,019.92 ± 68.99 mg/mL; TEAC: 52.70 ± 0.47 mg/mL); whereas tiliroside, isolated from *W. periplocifolia*, *H. crispa* and *S. micrantum* presented a low IC_50_ value (1.63 ± 0.86 mg/mL) compared to ascorbic acid in the TEAC assay.

## 1. Introduction

Reactive oxygen species (ROS), such as superoxide radicals, hydroxyl (OH) radicals and peroxyl radicals, are natural byproducts of the normal metabolism of oxygen in living organisms with important roles in cell signalling [1,2]. However, excessive amounts of ROS may be a primary cause of biomolecular oxidation and may result in significant damage to cell structure, contributing to various diseases, such as cancer, stroke, diabetes and degenerative processes associated with ageing [3,4]. Thus, antioxidants are important inhibitors of lipid peroxidation not only for food protection but also as a defense mechanism of living cells against oxidative damage [5]. Antioxidants have been shown to prevent the destruction of b-cells [6,7], and to prevent or inhibit oxidation processes in human body and food products [8].

Plant polyphenols with antioxidant capacity could scavenge reactive chemical species as well as minimise oxidative damage resulting from excessive light exposure. Some plant polyphenols are important components of both human and animal diets and they are safe to be consumed [9].

Food antioxidants such as α-tocopherol, ascorbic acid, carotenoids, amino acids, peptides, proteins, flavonoids and other phenolic compounds might also play a significant role as physiological and dietary antioxidants [10]. Natural antioxidants are known to exhibit a wide range of biological effects including antibacterial, antiviral, antiinflammatory, antiallergic, antithrombotic and vasodilatory activities [11].

Antioxidant capacity is widely used as a parameter for medicinal bioactive components. Various methods are currently used to assess the antioxidant activity of plant phenolic compounds. ABTS^•+^ or DPPH^•^ radical scavenging methods are common spectrophotometric procedures for determining the antioxidant capacities of components [12].

Assays based upon the use of DPPH^•^ and ABTS^•+^ radicals are among the most popular spectrophotometric methods for determination of the antioxidant capacity of food, beverages and vegetable extracts [13]. DPPH is a stable free radical that reacts with compounds that can donate a hydrogen atom. This method is based on the scavenging of DPPH through the addition of a radical species or an antioxidant that decolourizes the DPPH solution [14]. 

The Trolox equivalent antioxidant capacity assay (TEAC) is based on the ability of antioxidants to quench the long-lived ABTS radical cation, a blue/green chromophore with characteristic absorption at 734 nm, in comparison to butylated hydroxyanisole (BHA), butylated hydroxytoluene (BHT), α-tocopherol and Trolox, a water-soluble α-tocopherol analogue [12].

The Malvaceae family consists of 243 genus and 4,225 species [15]. In Brazil, there are 35 genus and 400 species [16]. The species of this family are utilized in folk medicine as emollients, antipyretics, anti-inflammatory, diuretic, arthritis, gastrointestinal disorders [17,18], snake bites [19] and asthma [20]. 

Due to the ethnobotanical importance of the family and the absence of studies that prove their antioxidant activity, the aim of this study was to evaluate the antioxidant activity, using DPPH radical scavenging activity and Trolox equivalent antioxidant capacity, and the total phenolic content of the crude extract (CE) and various phases depending on the species: ethyl acetate (EaF), aqueous (WtF), chloroform (CfF) hexane (HF), *n*-butanol (*n*-BF) or dichloromethane (DF) phases of plants of the Malvaceae family: *Sidastrum micranthum*, *Wissadula periplocifolia*, *Sida rhombifolia* and *Herissantia crispa*. The antioxidant activity of two flavonoids isolated from *Wissadula periplocifolia* and *Herissantia crispa*: kaempferol 3,7-di-O-α-L-rhamnopyranoside (lespedin) and kaempferol 3-O-β-D-(6*''*-E-p-coumaroil) glucopyranoside (tiliroside) was also analyzed. Tiliroside was also isolated from *Sidastrum micranthum*. 

## 2. Experimental

### 2.1. General

Ascorbic acid, 2,2*'*-azinobis-(3-ethylbenzothiazoline-6-sulphonic acid) (ABTS), 6-hydroxy-2,5,7,8-tetramethylchromane-2-carboxylic acid (Trolox), potassium persulfate, Folin-Ciocalteau’s reagent, 1,1-diphenyl-2-pycrylhydrazyl (DPPH) were purchased from Sigma Aldrich (St. Louis, MO, USA). Gallic acid and sodium carbonate were purchased from Fluka (Switzerland). All the chemicals were of analytical grade.

### 2.2. Plant

Leaves of the species under study where dried in an oven, at an average temperature of 40 °C, for 96 h. The dried leaves were macerated with ethanol (95%), for 72 h, at room temperature. The ethanol solution was concentrated under reduced pressure, at 50 °C, affording the crude extract (CE). The CE was partitioned in ethanol-H_2_O (7:3), extracted with hexane, chloroform, ethyl acetate or *n*-butanol to give the corresponding phases with crescent polarities: hexane (HF), chloroform (CF), ethyl acetate (EaF), *n*-butanol (nBF), water (WtF) of the species *Sidastrum micranthum* (A. St.-Hill.) Fryxell, *Wissadula periplocifolia* (L.) C. Presl and *Herissantia crispa* L. (Brizicky), and hexane (HF), dichloromethane (DF), ethyl acetate (EaF), *n*-butanol (nBF) and water (WtF) for *Sida rhombifolia.*

Kaempferol 3-*O*-β-D-(6*''*-E-*p*-coumaroyl)-glucopyranoside (tiliroside—500 mg) was isolated from the EaF phase of *W. periplocifolia* (L.) C. Presl [21], *S. micranthum* (A. St.-Hil.) Fryxell and *H. crispa* L. (Brizicky) ethanolic crude extract. The kaempferol was purified by successive Sephadex LH-20 column chromatography eluted with methanol [21,22,23]. Kaempferol 3,7-di-*O*-α-L-rhamnopyranoside (lespedin—200 mg) was isolated from the *n*-butanol phase (nBF) of the ethanolic crude extract of *W. periplocifolia* and *H. crispa* L. (Brizicky), using the same methodology [23]. 

**Figure 1:**
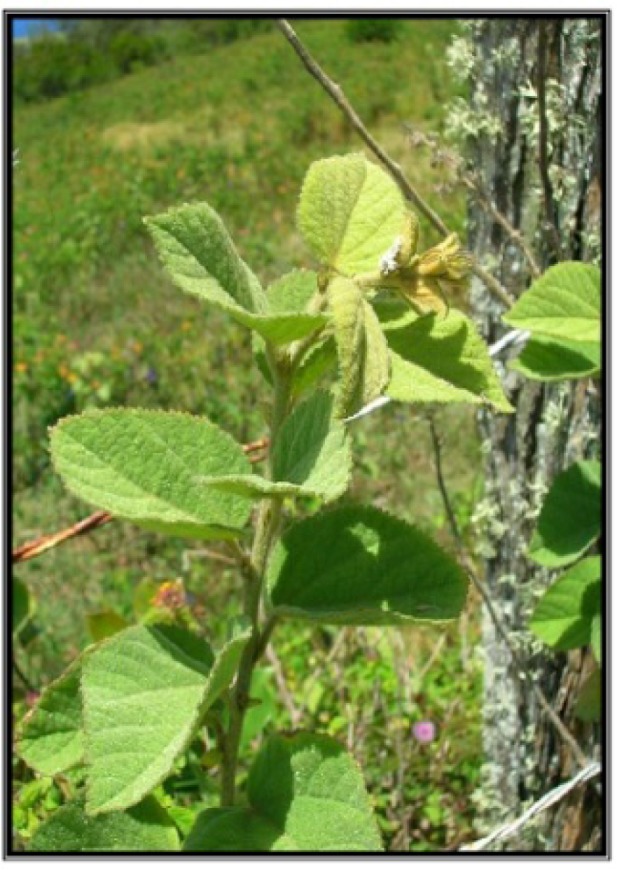
*Sidastrum micranthum* (A. St.-Hil.) Fryxell.

**Figure 2:**
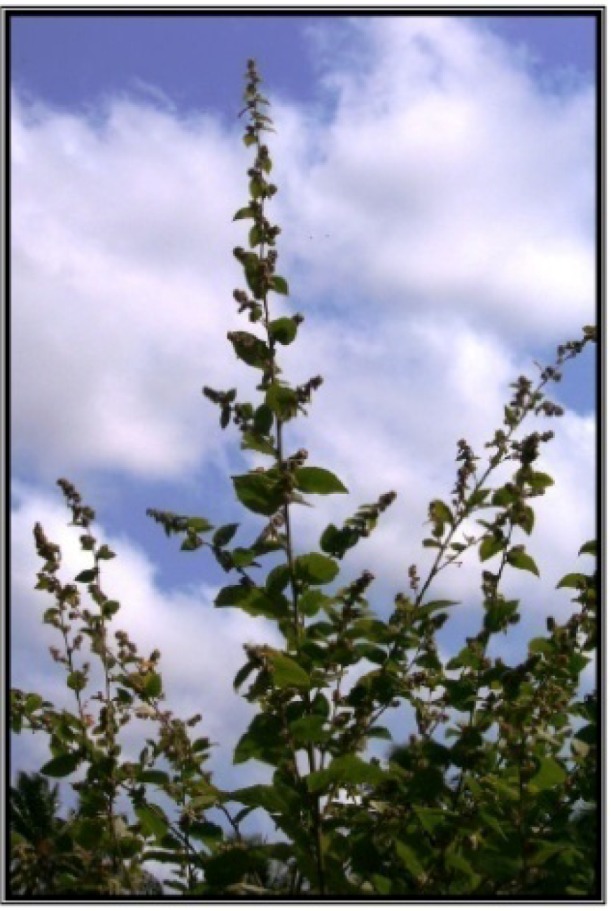
*Wissadula periplocifolia* (L.) C. Presl.

**Figure 3:**
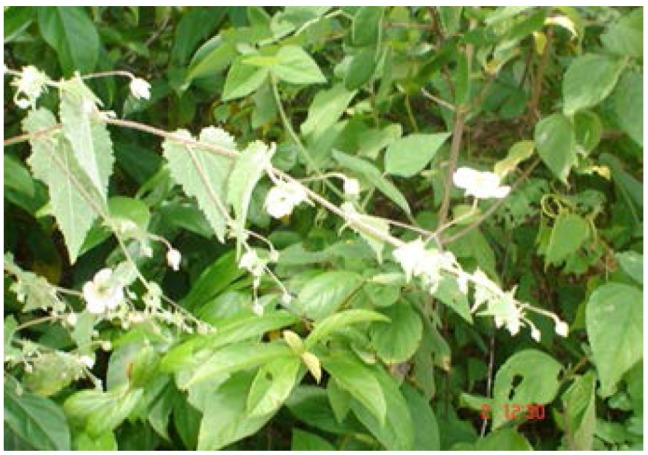
*Herissantia crispa* L. (Brizicky).

### 2.3. Determination of Total Phenolic Contents

The total phenolic content of the samples was determined using the Folin-Ciocalteau’s reagent as described by Gulcin *et al*. [24]. An aliquot of the samples, dissolved in ethanol, was mixed with Folin-Ciocalteau’s reagent (100 µL) and distilled water (3 mL) and mixed for 1 min. Sodium carbonate (300 µL, 15%) was added to the mix. The solution had its volume adjusted to 5 mL with distilled water. After 2 h, absorbance was measured at 760 nm. A standard curve was prepared using gallic acid with a concentration range from 0.5 to 25 µg/mL. Total phenolic content was expressed as mg gallic acid equivalents (GAE)/g of samples.

### 2.4. DPPH Radical Scavenging Activity Assay

The assay was performed according to Silva *et al.* [25]. An aliquot of the samples was mixed with DPPH solution (5 mL, 23.6 μg/mL in ethanol), followed by incubation of 30 min. The absorbance of each sample was read at 517 nm. Ascorbic acid (0.9, 1.9, 3.9, 4.9, 6.9 μg/mL) was used as positive reference. The percentage of scavenged DPPH was calculated using Equation (1):

DDPH scavenging activity = 100 × (*A*_c_ – *A*_s_)/*A*_c_ (1)

where *A*c is the absorbance of the control and *As* is the absorbance of the sample. IC_50_ values calculated denote the concentration of the sample required to decrease the absorbance at 517 nm by 50%. 

### 2.5. Trolox Equivalent Antioxidant Capacity Assay (TEAC)

The ABTS free radical-scavenging activity of each sample was determined according to the method described by RE *et al.* [26]. The radical cation ABTS^•+^ was generated by persulfate oxidation of ABTS. A mixture of ABTS (7.0 mM) and potassium persulfate (2.45 mM) was allowed to stand overnight at room temperature in the dark to form radical cation ABTS^•+^, 12–16 h prior to use. A solution was diluted with ethanol and absorbance measured, at 734 nm. An aliquot of each sample was mixed with the solution of the radical cation ABTS^•+^ (5 mL), and the decrease of absorbance was measured at 734 nm after 10 min. Trolox (1.1, 1.7, 2.3, 2.9, 3.5 μg/mL) was used as positive reference. IC_50_ values calculated denote the concentration of the sample required to decrease the absorbance at 517 nm by 50%.

All experiments were performed in triplicate. The DPPH and TEAC data were expressed as IC_50_ (mg/mL). Total phenolic content was expressed as mg gallic acid equivalents (GAE)/g. Linear regressions were performed to indicate the relationship between the total phenolic contents and data from the antioxidant assays.

## 3. Results and Discussion

The results of total phenolic contents and the antioxidant activity of the EEB and phases of *Sidastrum micranthum* are shown in Table 1. The ethyl acetate (EaF) phase of this species, had the highest content of phenolic compounds (177.44 ± 16.21 mg·GAE/g) and the highest TEAC (IC_50_ = 2.267 ± 0.377), showing a better result than the Trolox assay (IC_50_ = 3.02 ± 0.014). However, for the DPPH assay, the chloroform (CfF) phase showed better antioxidant activity (IC_50_ = 24.7 ± 0. 306). 

The results of the antioxidant assays of *Wissadula periplocifolia* are shown in Table 2. The EaF phase had the highest content of phenolic compounds (260.46 ± 5.74), as well as the best antioxidant activity in the DPPH assay (IC_50_ = 20.52 ± 0.16). The TEAC assay, the CfF phase showed better antioxidant activity (IC_50_ = 23.98 ± 0.03) compared to the other phases. This discrepancy in total antioxidant activity values depending on the method used indicates that both assays determine different aspects of the antioxidant capacity. Different radicals and mechanisms of reaction are occurring [27].

**Table 1:** Total polyphenol contents, DPPH radical scavenging activity and Trolox equivalent antioxidant capacity (TEAC) of *Sidastrum micranthum.*

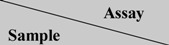	Total polyphenol contents (mg·GAE/g)	DPPH (IC_50_—mg/mL)	TEAC (IC_50_—mg/mL)
CE	39.37 ± 2.54	125.733 ± 0.291	35.543 ± 0.954
EaF	177.44 ± 16.21	147.533 ± 2.786	2.267 ± 0.377
WtF	22.83 ± 2.14	111.123 ± 0.963	35.503 ± 0.358
CfF	107.29 ± 4.17	24.7 ± 0.306	10.053 ± 0.132
HF	17.59 ± 1.74	100.83 ± 2.478	34.447 ± 0.468
*n*-BF	50.88 ± 2.78	159.267 ± 6.02	24.687 ± 1.054
Positive reference	-	3.124 ± 0.023	3.02 ± 0.014

Crude Extract (CE), ethyl acetate (EaF), aqueous (WtF), chloroform (CfF), hexane (HF), *n*-butanol (*n*-BF), dichloromethane (DF). Positive reference DPPH: ascorbic acid. Positive reference TEAC: Trolox.

**Table 2:** Total polyphenol contents, DPPH radical scavenging activity and Trolox equivalent antioxidant capacity (TEAC) of *Wissadula periplocifolia.*

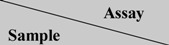	Total polyphenol contents (mg·GAE/g)	DPPH (IC_50_—mg/mL)	TEAC (IC_50_—mg/mL)
CE	45.68 ± 0.58	114.28 ± 0.60	75.42 ±0.44
EaF	260.46 ± 5.74	20.52 ± 0.16	45.71 ± 0.07
WtF	26.41 ± 1.62	129.11 ± 0.64	83.15 ± 2.33
CfF	118.64 ± 0.94	54.92 ± 0.52	23.98 ± 0.03
HF	23.89 ± 0.89	151.18 ± 2.20	92.78 ± 0.52
*n*-BF	34.02 ± 1.48	91.28 ± 0.43	49.90 ± 1.04
Positive reference	-	3.124 ± 0.023	3.02 ± 0.014

Crude Extract (CE), ethyl acetate (EaF), aqueous (WtF), chloroform (CfF), hexane (HF), *n*-butanol (*n*-BF), dichloromethane (DF). Positive reference DPPH: ascorbic acid. Positive reference TEAC: Trolox.

The EaF of the *Sida rhombifolia* had the highest content of phenolic compounds (88.311 ± 2.660 mg·GAE/g) and the best antioxidant activity for the DPPH and TEAC assay (IC_50_ = 70.503 ±1.629 and 20.580 ± 0.271, respectively), as shown in Table 3.

**Table 3:** Total polyphenol contents, DPPH radical scavenging activity and Trolox equivalent antioxidant capacity (TEAC) of *Sida rhombifolia.*

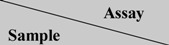	Total polyphenol contents (mg·GAE/g)	DPPH (IC_50_—mg/mL)	TEAC (IC_50_—mg/mL)
CE	38.22 ± 0.43	115.91 ± 0.15	75.70 ± 1.45
EaF	88.31 ± 2.66	70.50 ±1.62	20.58 ± 0.27
WtF	3.01 ± 0.32	126.46 ± 1.62	67.67 ± 0.38
DF	69.75 ± 1.87	87.99 ± 0.26	32.32 ± 0.79
HF	16.50 ± 0.19	180.21 ± 4.72	112.02 ± 0.29
*n*-BF	22.75 ± 1.66	111.88 ± 1.50	58.46 ± 0.47
Positive reference	-	3.124 ± 0.023	3.02 ± 0.014

Crude Extract (CE), ethyl acetate (EaF), aqueous (WtF), chloroform (CfF), hexane (HF), *n*-butanol (*n*-BF), dichloromethane (DF). Positive reference DPPH: ascorbic acid. Positive reference TEAC: Trolox.

The CfF phase of *Herissantia crispa*, had the highest content of phenolic compounds (142.397 ± 0.555 mg·EAG/g), and the best antioxidant activity for DPPH assay (IC_50_ = 61.52 ± 0.458) and TEAC assay (IC_50_ = 27.127 ± 0.567) (Table 4).

**Table 4:** Total polyphenol contents, DPPH radical scavenging activity and Trolox equivalent antioxidant capacity (TEAC) of *Herissantia crispa.*

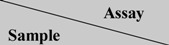	Total polyphenol contents (mg·GAE/g)	DPPH (IC_50_—mg/mL)	TEAC (IC_50_—mg/mL)
CE	56.25 ± 0.83	120.06 ± 3.10	80.84 ± 1.04
EaF	87.07 ± 0.50	107.09 ± 2.22	83.35 ± 0.08
WtF	73.68 ± 3.51	173.23 ± 0.93	116.02 ± 1.03
CfF	142.39 ± 0.55	61.52 ± 0.45	27.12 ± 0.56
HF	45.76 ± 2.39	255.05 ± 3.97	150.20 ± 3.30
*n*-BF	63.21 ± 2.40	97.93 ± 0.68	91.77 ± 0.74
Positive reference	-	3.124 ± 0.023	3.02 ± 0.014

Crude Extract (CE), ethyl acetate (EaF), aqueous (WtF), chloroform (CfF), hexane (HF), *n*-butanol (*n*-BF), dichloromethane (DF). Positive reference DPPH: ascorbic acid. Positive reference TEAC: Trolox.

Only David *et al.* [28] have studied the methanolic extract of *Herissantia crispa,* reporting an IC_50_ value for the DPPH assay of 3.9 mg/mL. However, other phases were not studied.

The Folin-Ciocalteu method, generally used to assay the total phenolic compound content also measures the total reducing capacity of a sample. Total phenolics generally correlate with redox and antioxidant capacities as measured by the TEAC or DPPH methods [29,30]. Many studies indicate a linear relationship between total phenolics and antioxidant activity [31,32,33]. A direct correlation between the three methods, in all species, was demonstrated by linear regression analysis. As shown in this study, there is a strong correlation between total polyphenol contents and the antioxidant activity (r^2^ = 0.929) of *Sidastrum micranthum*; as well as *Wissadula periplocifolia* (r^2^ = 0.814) samples. 

Liu *et al*. [34] showed that the correlations of total polyphenol content against the antioxidant activity based on the DPPH assay, TEAC assay, and FRAP (ferric ion reducing antioxidant power) assay of *Ilex kudingcha* were satisfactory (r > 0.812). The results of that work indicated that polyphenols in kudingcha extracts are largely responsible for the antioxidant activities.

Phenolic compounds are ubiquitous bioactive compounds and a diverse group of secondary metabolites universally present in higher plants [35]. The Folin-Ciocalteu phenol reagent is used to obtain a crude estimate of the amount of phenolic compounds present in an extract. However, the assay has been shown nonspecific not only to polyphenols but to any other substance that could be oxidised by the Folin reagent as reported by various researchers, the poor specificity of the assay [36,37]. This statement may explain the low correlation between total polyphenol contents and the antioxidant activity of *Sida rhombifolia* and *Herissantia crispa* (r^2^ = 0.618; r^2^ = 0.46, respectively). It is suggested, that non-phenolic substances are responsible for antioxidant activity in this species. This lack of relationship is in agreement with Anagnostopoulou *et al*. [38] who reported a r^2^ value of 0.42 between TPC and DPPH for extract obtained from sweet orange peel.

Flavonoids are polyphenolic compounds with low molecular mass, found in leguminous, fruits, flowers, and leaves [39], having several biological activities, for example, anti-inflammatory [40,41] and antioxidant action [42]. Some flavonoids isolated from Malvaceae species [43,44], were: 3,7,3′,4′-tetrahydroxyflavone (quercetin); 5,7-dihydroxy-4′-methoxyflavone (acacetin); 7,4′-di-O-methyl-isoscutellarein; 5,8-dihydroxy-7,4′-dimethoxyflavone; 5,4′-dihydroxy-7-methoxyflavone (genkwanin) [22]; 5,4′-dihydroxy-3,7-3′-trimethoxyflavone (pachypodol); luteolin 7-O-β-D-glucopyranoside (cinaroside), 5,7,4′ trihydroxyflavone (apigenin); 5,7,3′,4′-tetrahydroxyflavone (luteolin) [F]; kaempferol-3-O-β-D-(6′′-*E*-*p*-coumaroyl)glucopyranoside (tiliroside) [21,22,23,45,46,47] and kaempferol 3,7-di-O-α-L-rhamnoside (lespedin). 

Lespedin showed a high IC_50_ value for the DPPH (IC_50_ = 1,019.92 ± 68.99) and TEAC assay (IC_50_ = 52.70 ± 0.47) (Table 5), which means that this flavonoid is not the main substance responsible for the antioxidant activity of the EaF phase of *Wissadula periplocifolia.* Tiliroside also showed a high IC_50_ value for the DPPH assay (IC_50_ = 219.31 ± 9.62), therefore, it cannot be responsible for the antioxidant activity of the EaF phase. However, for the TEAC assay, it exhibited a low IC_50_ value (IC_50_ = 1.63 ± 0.86), and may contribute to the antioxidant activity of the Eaf phase of *Wissadula periplocifolia* and *Sidastrum micranthum*.

**Table 5:** DPPH radical scavenging activity and Trolox equivalent antioxidant capacity (TEAC) of Tiliroside e Lespedin.

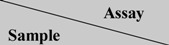	DPPH (IC_50_—mg/mL)	TEAC (IC_50_—mg/mL)
Lespedin	1,019.92 ± 68.99	52.70 ± 0.47
Tiliroside	219.31 ± 9.62	1.63 ± 0.86
Positive reference	3.124 ± 0.023	3.02 ± 0.014

Positive reference DPPH: ascorbic acid. Positive reference TEAC: Trolox.

A study performed by Babbar *et al*. showed that phenolic compounds alone are not fully responsible for the antioxidant activity of plants. Other constituents such as ascorbates, reducing carbohydrates, tocopherols, carotenoids, terpenes, and pigments as well as the synergistic effect among them could possibly contribute to the total antioxidant activity [48]. 

## 4. Conclusions

In conclusion, species the Malvaceae family have a high content of phenolic compounds and a good antioxidant activity, therefore they can be used to treat several diseases in which there is an increase in free radical production. However, non-phenolic substances can be responsible for the antioxidant activity of *Herissantia crispa*. Therefore, further studies are needed to identify which phenolic compounds are responsible for the antioxidant activity of the species, and assess the way in which the phenolic substances contribute to this activity. Additional *in viv*o antioxidant assays are needed to confirm the potential use of these species in disease treatment. 
